# Acute urinary retention during menstruation: Answers

**DOI:** 10.1007/s00467-020-04646-9

**Published:** 2020-06-18

**Authors:** Hannah Jeffery, Swati Jha

**Affiliations:** 1 Department of Paediatric Surgery, Sheffield Children’s Hospitals, Sheffield, UK; 2 Department of Urogynaecology, Sheffield Teaching Hospitals, Level 4 Jessop Wing, Tree Root Walk, Sheffield, S10 2SF UK

**Keywords:** Puberty, Hemivagina, Uterine didelphys, Renal agenesis, Müllerian duct anomaly

## Answers

1. OHVIRA (obstructed hemivagina and ipsilateral renal anomaly) syndrome. The usual presentation is with menstrual problems with worsening dysmenorrhoea, lower abdominal pain and sometimes a pelvic mass.
2. The condition is managed by removal of the vaginal septum causing the obstruction.
3. Potential complications include pelvic abscess, pelvic adhesions and endometriosis.

## Discussion

Acute urinary retention during menstruation is a rare occurrence in a child. Possible causes may be broadly classified into obstructive, neurological, pharmacological and psychogenic categories. It needs urgent investigation. In this case, there was an obvious obstructive cause.

**Fig. 1:**
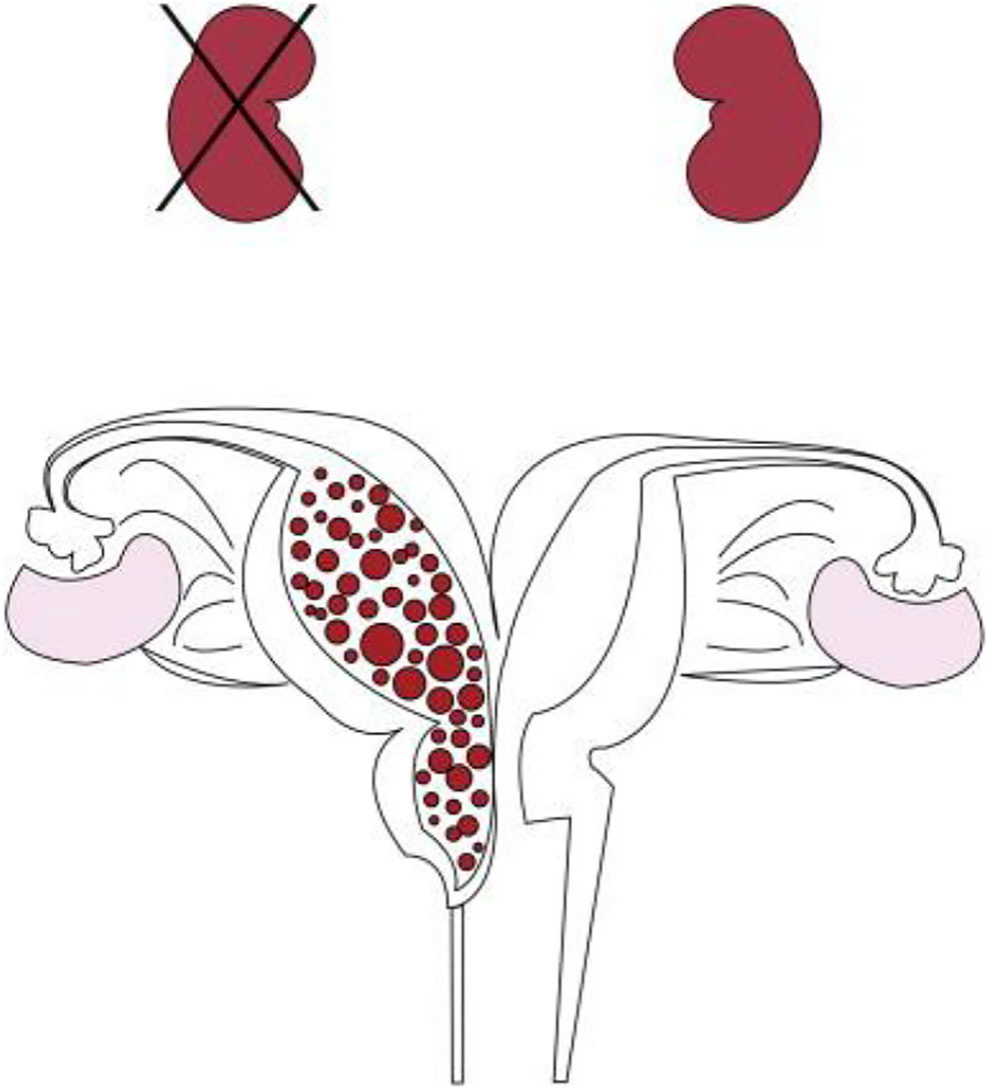
Graphical representation of the triad of OHVIRA syndrome

OHVIRA (obstructed hemivagina and ipsilateral renal anomaly) is a rare condition first reported in 1925 and subsequently described in detail by Herlyn, Werner and Wunderlich after whom it was initially named HWW syndrome. It is characterised by the triad of uterine didelphys, obstructed hemivagina and ipsilateral renal anomaly (Fig. 1). Delays in diagnosis are common due to normal onset of puberty and menstruation. Patients usually present with progressive dysmenorrhoea and non-specific lower abdominal pain, though other presentations such as urinary retention, as in this case, and pelvic mass are possible. The incidence is unknown but has been quoted at 0.1% and 3% [1], and the etiology is poorly understood. There are a range of phenotypic variables; hence, when patients present with dysplastic or absent kidneys, an attempt to establish uterovaginal anomalies should be made to allow early recognition and management. However, because the presentation is variable, prompt recognition can be difficult. Though renal agenesis is the classic presentation, other anomalies include renal dysplasia and ectopic ureters [2]. As well as renal anomalies ipsilateral to the obstructed hemivagina, contralateral renal anomalies have been reported in up to 50% [2, 3]. Cervical atresia and a double cervix with hemicervical obstruction with a rudimentary hemiuterus may present as OHVIRA syndrome. Renal physicians need to be aware of this condition to allow appropriate referral to a paediatric gynaecologist.

Investigation of this condition is best done by MRI imaging. This is because the obstructed hemivagina distorts the anatomy, making it difficult to assess with an ultrasound scan. All cases should be managed in a tertiary centre where there is adequate expertise in managing Müllerian duct anomalies and multidisciplinary approach to care. In the absence of acute symptoms surgical treatment can be elective with initial suppression of menstruation. There is no requirement for GnRH analogues for menstrual suppression as this can be achieved with oral preparations of progesterone (norethisterone, 5 mg BD). Resection of the septum under direct visualisation is the definitive management. The vaginal septum is usually easy to identify as it is distended. Making a small hole in the septum is inappropriate as this promotes ascending infections. This surgery should therefore be undertaken by a paediatric gynaecologist with expertise in vaginal surgery.

There is a risk of incomplete excision of the septum and the need for further surgery. There is also an increased risk of endometriosis (due to retrograde menstruation) and miscarriage/preterm labour in later life, and there has been a case report of conception occurring in the obstructed uterine horn. Cervical screening from both cervices is required.

Variants of OHVIRA have been recognised where the septum has a fenestration resulting in only a partial obstruction as reported in a series by Fedele [4]. Occasionally, the septum is thin and retained menstrual contents can cause it to perforate resulting in a communication between the two vaginas [5]. This can also predispose to ascending bacterial infections with development of a pyometra or a pelvic infection which may require emergency excision of the septum.

In most patients with OHVIRA, the septum is thick and limits the ability of the hemivagina to distend, resulting in retrograde bleeding causing endometriosis. Endometriosis has been reported in 17–35% of patients with this condition [5–7]. This can be avoided by timely decompression.

In patients with OHVIRA, ovarian function is normal. The impact of this condition on fertility is not well known, but problems may arise due to endometriosis, development of pelvic abscess and pelvic adhesions. There are case reports of pregnancy occurring in the obstructed hemiuterus [8]. Early diagnosis and removal of the obstructing septum can prevent complications and allow preservation of fertility in most patients. Where the condition is complicated by cervical atresia, surgery may be more complex.

Kidney function should be established and monitored as deterioration can occur around puberty due to rapid somatic growth. There are theoretical risks of hypertension, proteinuria and chronic kidney disease in later life. Patients should be advised to avoid factors that may impact on kidney function such as NSAIDS.
